# In silico prediction of bioequivalence of atorvastatin tablets based on GastroPlus™ software

**DOI:** 10.1186/s40360-023-00689-4

**Published:** 2023-11-28

**Authors:** Lu Wang, Jinliang Chen, Wenjun Chen, Zourong Ruan, Honggang Lou, Dandan Yang, Bo Jiang

**Affiliations:** Center of Clinical Pharmacology, The Second Affiliated Hospital, Zhejiang University School of Medicine, 88 Jiefang Road, Hangzhou, 310009 Zhejiang China

**Keywords:** Atorvastatin, In silico modeling, GastroPlus, Bioequivalence

## Abstract

The prediction of intestinal absorption of various drugs based on computer simulations has been a reality. However, in vivo pharmacokinetic simulations and virtual bioequivalence evaluation based on GastroPlus™ have not been found. This study aimed to simulate plasma concentrations with different dissolution profiles and run population simulations to evaluate the bioequivalence of test and reference products of atorvastation using GastroPlus software. The dissolution profiles of the reference and test products of atorvastatin (20 mg tablets), and clinical plasma concentration-time data of the reference product were used for the simulations. The results showed that the simulated models were successfully established for atorvastatin tablets. Population simulation results indicated that the test formulation was bioequivalent to the reference formulation. The findings suggest that modelling is an essential tool to demonstrating the possibility of pharmacokinetic and bioequivalence for atorvastatin. It will contribute to understanding the potential risks during the development of generic products.

## Introduction

In vivo bioequivalence (BE) studies are pivotal to ensure equivalence between a generic and an innovator product. However, highly variable drugs have a risk of failure when the BE studies were performed directly on subjects [1, 2]. A wrong decision will set you back severely in terms of time, money, and getting into market. Therefore, it is crucial to consider incorporating appropriate technology to help with the early assessment of potential BE risk before conducting a clinical study.

Over the past decades, the computer simulation has rapidly become an integral tool in drug discovery and development [3–5]. GastroPlus is a mechanistically based software that integrates various physiologic and drug-property parameters. It has been demonstrated to predict PK behavior accurately and allow simulation for different demographics, thus supporting decisions at various stages of the drug development process [6–10]. GastroPlus has also been applied to predict oral absorption of immediate-release and extended-release products containing different dissolution profiles, which will contribute to the decision on the best condition of dissolution test and BE outcome [11–13].

Atorvastatin, a synthetic reversible inhibitor of 3-hydroxy-3-methylglutaryl- coenzyme A (HMG-CoA) reductase, is indicated for the treatment of hyperlipidemias. when administered orally as a calcium salt, it is rapidly and completely absorbed in the intestine in vivo, with maximum plasma concentration occurring in the range of 0.5 to 1.5 hours [14–17]. A non-linear increase in systemic concentration of atorvastatin became evident at doses of 40 mg and above [14]. Moreover, atorvastatin is a highly variable drug and undergoes extensive first-pass metabolism in the walls of gut and liver, resulting in low oral bioavailability [14]. Although gut wall metabolism significantly contributes to the first-pass effect [14], hepatic metabolism is the primary route of the elimination of atorvastatin.

Studies have reported the pharmacokinetics and bioequivalence of generic and innovator atorvastatin [15, 18, 19], as well as physiologically-based pharmacokinetic modeling of disposition and drug-drug interactions [20–22]. However, no previous research has described in vivo pharmacokinetic simulations and virtual BE evaluation of atorvastatin using GastroPlus. The study aimed to predict plasma concentrations of atorvastatin tablets, asses the effects of in vitro dissolution behaviour on in vivo absorption outcome, and evaluate the BE of two atorvastatin products using the GastroPlus software.

## Materials and methods

### Materials

Atorvastatin (20 mg) tablets were obtained from Slinopep-Alisino Biopharmaceutical Co., Ltd. (Test) and Pfizer Pharmaceuticals Ltd (Reference).

### Software

GastroPlus (Version 9.8; Simulations Plus, Inc., Lancaster, California, USA) was used for all the in vivo pharmacokinetic simulations of atorvastatin tablets. ADMET Predictor® (Version 10.2, Simulations Plus, Inc., CA, United States), a module in GastroPlus, was used to predict the physicochemical parameters of atorvastatin. Additionally, we use PKPlus, an optional module in GastroPlus, to perform compartmental modelling using oral plasma concentration-time data and generate PK parameters for simulations.

### Construction and verification of in silico model

We utilized GastroPlus to conduct all in vivo pharmacokinetic simulations. The input parameters of GastroPlus for atorvastatin to build the model are shown in Table 1. Human P_eff_ was obtained from Caco-2 data for the reference drug [23]. Log P, pKa, and fraction unbound in plasma (f_u_) were projected from the predicted values of ADMET software. Furthermore, we optimized the blood to plasma ratio (R_bp_) and adjusted the coefficients in absorption scale factor (ASF) to fit the observed data better. We obtained the in vivo data for the oral administration of 20 mg and 40 mg atorvastatin tablets from a completed PK study and literature [16], respectively. The completed PK study has been approved by the Human Subject Research Ethics Committee of the Second Affiliated Hospital School of Medicine, Zhejiang University before the study.

**Table 1:** Input parameters used in GastroPlus™ to simulate plasma concentrations

Parameters	Value	Data Source
Molecular weight	558.65	GastroPlus Predicted
*Log* P	4.434	GastroPlus Predicted
*pK*a	4.71	GastroPlus Predicted
Rbp	7.61	Optimized
Fu	0.0504	GastroPlus Predicted
Caco-2 permeability (cm/s×10^− 6^)	2.08	Ref. [29]
P_eff_ (cm/s×10^− 4^)	1.536	GastroPlus Predicted based on Caco-2 data

Rbp, blood to plasma ratio; Fu, fraction unbound in plasma; P_eff_, effecve in vivo permeability

### In vitro dissolution study and prediction of in vivo performance

The test and the reference formulations dissolution tests were determined using a USP type II dissolution apparatus (paddle) with a rotational speed of 50 rpm. Three dissolution media were used, each containing of hydrochloric acid (pH 1.2), sodium acetate buffer (pH 4.5), and potassium phosphate buffer (pH 6.8), respectively. The volume of dissolution media was 900 mL. Samples were taken at 5, 10, 15, 20, 35, 45, and 60 min. The collected samples were filtered and determined using ultraviolet-visible spectrophotometry.

The in vitro dissolution profiles of the reference formulation in three pH media were directly loaded in a silico model, and “CR: dispersed tablet” was selected as the dosage form in GastroPlus. Combining in vitro dissolution curves, the human plasma concentration-time profiles were simulated.

### Population simulation

A population simulation was conducted to predict the outcome of a pivotal BE study. The population simulator will run a series of simulations with different simulated subjects. Each different simulated subject has a random set of physiological and pharmacokinetic parameters to imitate the variances of ADME. The number of virtual subjects chosen for simulation was 46. A virtual crossover trial was conducted by loading the same selected population for test and reference formulations. The default population parameter values in GastroPlus were used in the simulations. Based on previous clinical data, the coefficient of variation (CV) for C_max_ and AUC_0 − t_ was changed to 40% and 15%, respectively. The geomean ratio (GMR) and 90% confidence interval (CI) were automatically calculated in GastroPlus. The test formulation was considered bioequivalence to the reference formulation if the 90% CIs for C_max_, AUC_0 − t_, and AUC_0−∞_ were within the 80.00–125.00% range. To evaluate the reproducibility of this simulation, the in silico BE study simulation was repeated 10 times.

## Results

### Oral absorption model for prediction of PK

The input parameters of GastroPlus for atorvastatin model are shown in Table 1. After loading the oral plasma concentrations of the atorvastatin (20 mg tablets) in the Gastroplus software, PKPlus calculated the most appropriate compartmental model. The compartmental model data calculated by the PKPlus module is shown in Table 2. Due to the highest R^2^ and lowest AIC value, the three-compartmental model presented the best fitting ability. The PK parameters predicted using the three-compartmental model are shown in Table 3.

**Table 2:** T_1/2_, R^2^, and AIC for compartmental models

Compartmental Models	T_1/2_ (h)	R^2^	AIC
One-compartmental	7.261	0.7487	-36.06
Two-compartmental	11.55	0.8531	-53.58
Three-compartmental	18.82	0.9249	-60.99

**Table 3:** PK parameters from the three-compartmental model

Parameter	Value
Clearance, CL (L/h)	358.3
Central compartment volume, Vc (L)	16.02
Elimination half-life, T_1/2_ (h)	18.82
Distribution rate constant from C1 to C2, K12 (h^− 1^)	1.2677
Distribution rate constant from C2 to C1, K21 (h^− 1^)	0.65964
Distribution rate constant from C1 to C3, K13 (h^− 1^)	0.07081
Distribution rate constant from C3 to C1, K31 (h^− 1^)	0.04706

**Fig. 1:**
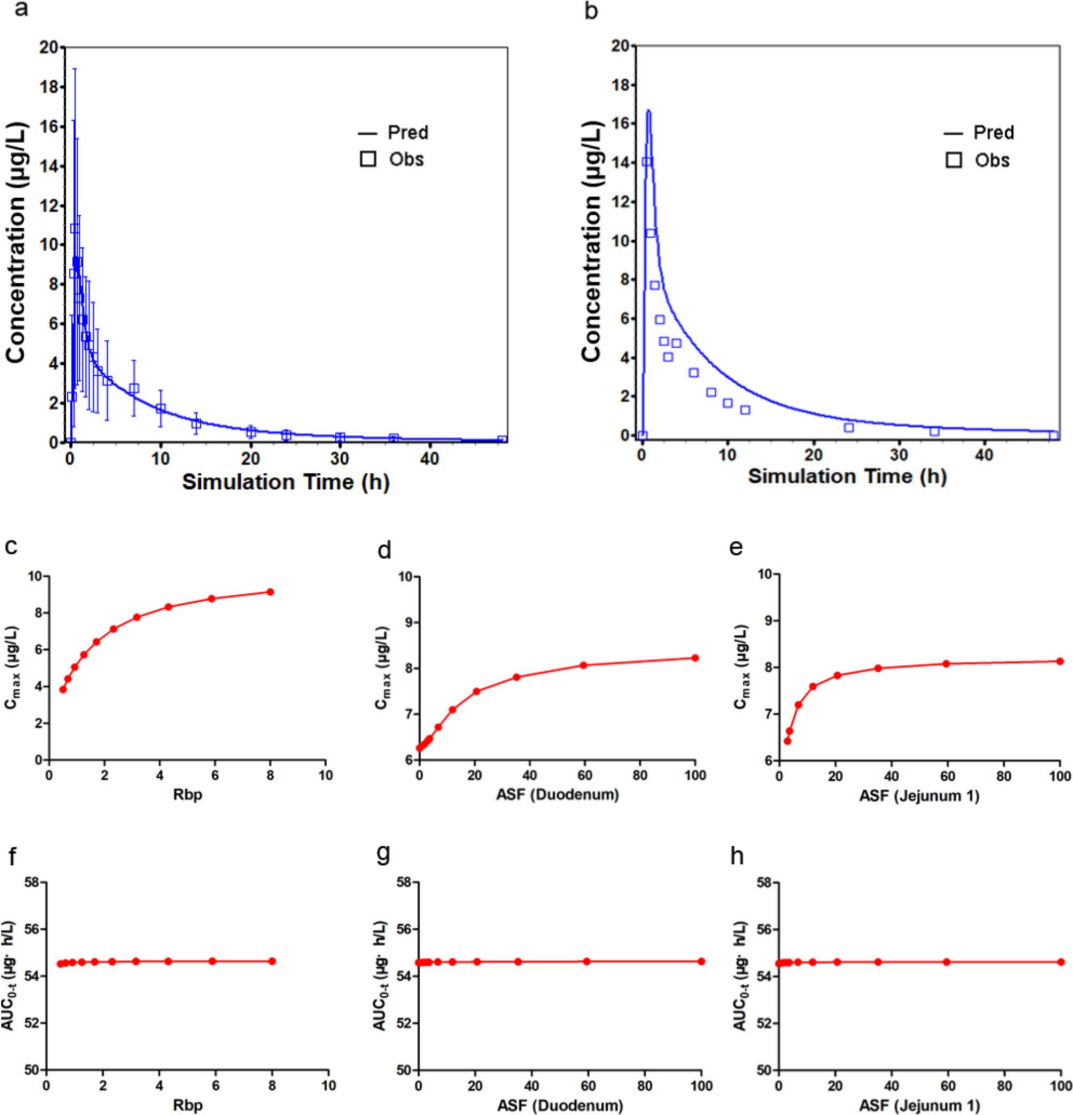
Simulated and observed plasma concentration-time profiles (**a**, 20 mg; **b**, 40 mg atorvastatin) and parameter sensitivity analysis on C_max_(**c-d**) and AUC_0 − t_(**e-f**)

GastroPlus was used to simulate plasma concentration-time curves after the oral administration of atorvastatin tablets at doses of 20 mg and 40 mg. We compared the predicted results with the observed data and found that the predicted/observed ratios were within approximately 2-fold for C_max_ and AUC, as shown in Fig. 1a, b, and Table 4. These results indicated that the model was able to reasonably predict the observed data. In fact, parameter sensitivity analysis (PSA) was used to assess the effect of input parameters on the rate and degree of drug absorption during model construction. As shown in Fig. 1c-e, R_bp_, duodenum, and jejunum ASF showed a positive correlation with C_max_. However, changes in R_bp_, duodenum, and jejunum ASF had no significant effect on AUC_0 − t_ (Fig. 1f-h).

**Table 4:** Simulated and observed pharmacokinetic parameters after oral administration of 20 mg and 40 mg tablets

Parameters	Atorvastatin 20 mg				Atorvastatin 40 mg		
	Obs	Pred	Pred/Obs		Obs	Pred	Pred/Obs
C_max_ (µg/L)	10.81	9.44	0.87		14.06	16.75	1.19
AUC_0–t_ (µg·h/L)	53.59	52.93	0.99		61.74	94.96	1.54
AUC_0–∞_(µg·h/L)	55.56	55.51	1.00		61.77	99.59	1.61

Obs: Observed data; Pred: Predicted data

### In vitro dissolution behavior and prediction of in vivo performance

**Fig. 2:**
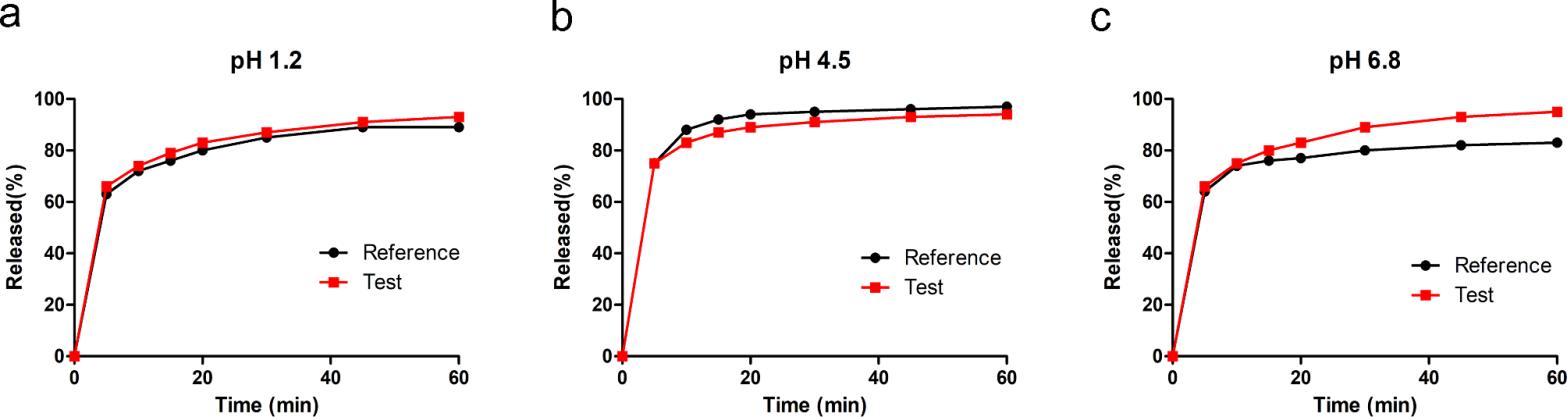
In vitro dissolution profiles of reference formulation at pH 1.2 (**a**), pH 4.5 (**b**) and pH 6.8 (**c**) for atorvastatin. Data are presented as mean, n = 6

Various dissolution media were evaluated to identify the discriminatory power of in vitro dissolution methods of atorvastatin. The dissolution profiles for the two atorvastatin formulations in different pH media are shown in Fig. 2. In pH 4.5 media, both the reference and test formulations nearly completely dissolved (> 85%) within 15 min. However, in pH 1.2 and pH 6.8 media, the dissolved reference and test formulations did not reach 85% of the total amount within 15 min. Despite this, the reference and test formulations exhibited similar dissolution profiles in all three media.

**Fig. 3:**
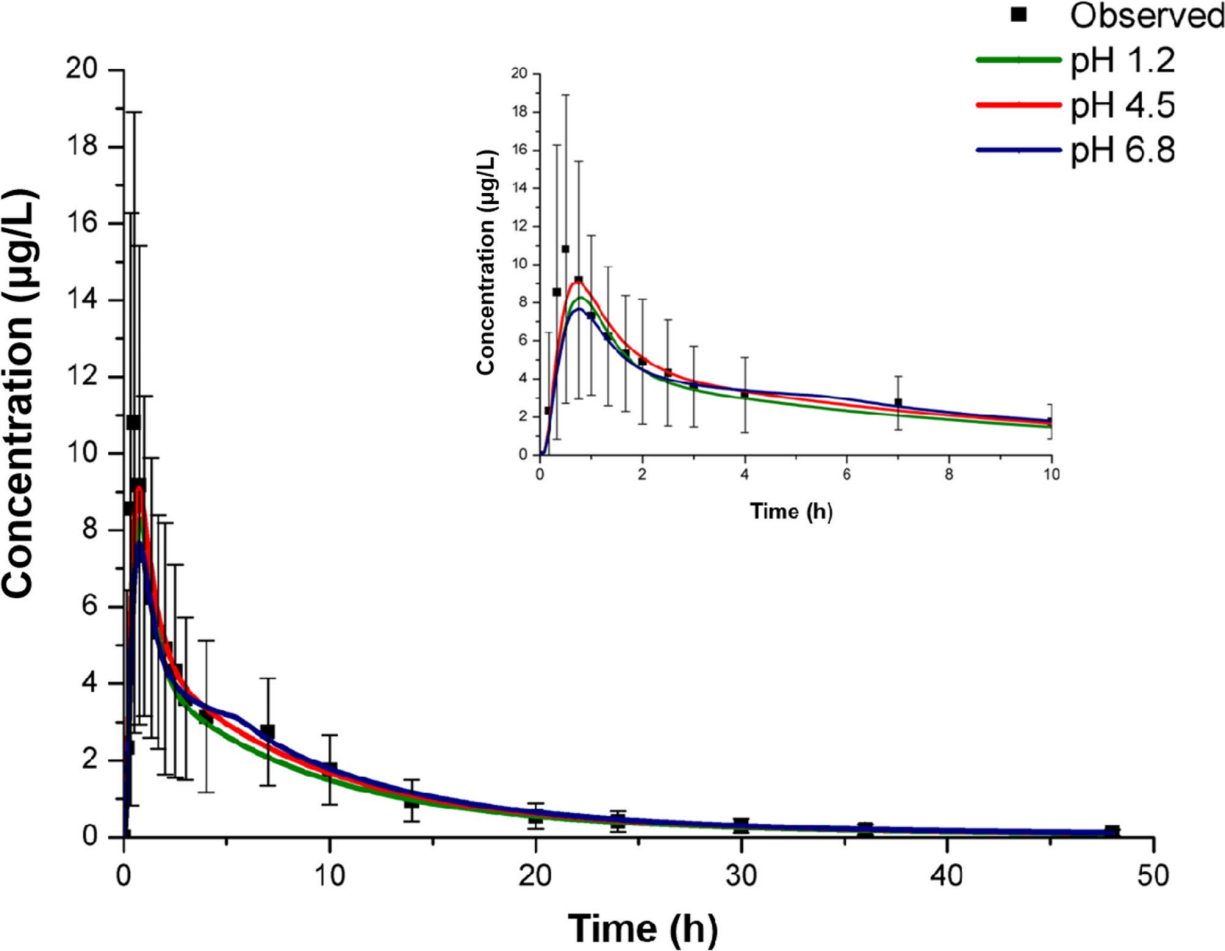
The dissolution and absorption correlation of atorvastatin tablets based on dissolution data; error bars represent the standard deviation (data are shown as mean ± SD)

To determine which dissolution media would capture the in vivo behaviour more accurately, the in vitro dissolution profiles of the reference formulation were directly loaded in the silico model. As shown in Fig. 3, high correlation (R^2^>0.85) and similar pharmacokinetic characteristics were observed in pH 1.2, pH 4.5, and pH 6.8 media compared to the observed value. However, the correlation for the predicted and observed plasma concentration curve in pH 4.5 media was higher, and the predicted C_max_ was more accurate than that predicted in the other two dissolution media. Therefore, the dissolution profiles of the pH 4.5 media were used to carry out population simulations and further assess the bioequivalence of the two formulations.

### Population simulations

**Fig. 4:**
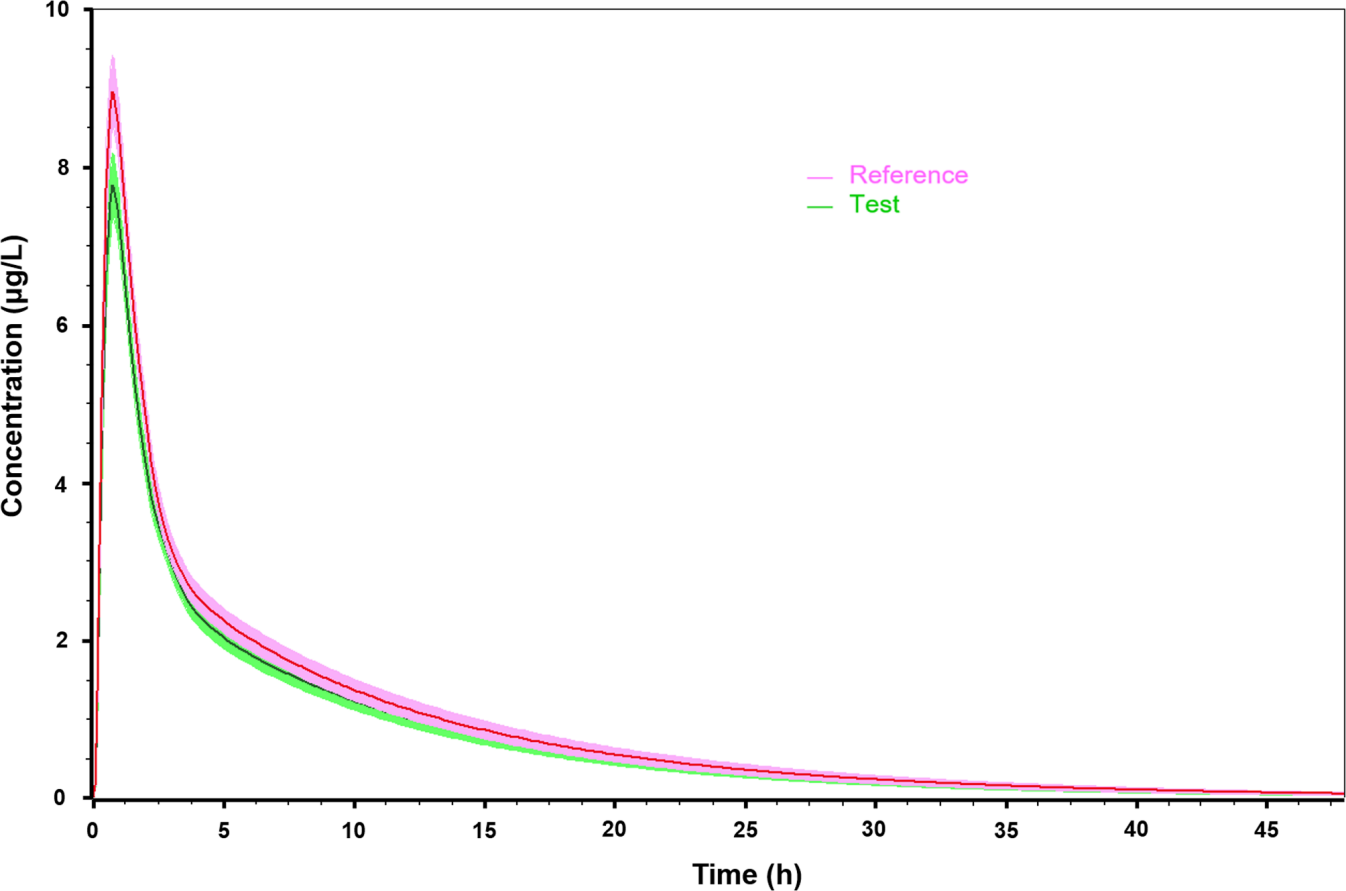
Outputs of representative virtual BE simulations in fasted states

Outputs of representative virtual BE results of 10 trials are presented in Fig. 4; Table 5, respectively. The test formulation was judged as bioequivalent in seven trials compared to the reference formulation. Only three simulation trials showed non-BE.

**Table 5:** Population simulation results of 20 mg atorvastatin tablets

Parameters	GeoMean T/R	90%CI		
C_max_ (µg/L)	89.34 (83.11, 95.26)	82.70 (75.92, 88.79)	to	96.51 (90.98, 102.48)
AUC_0–∞_(µg·h/L)	90.58 (86.92, 93.43)	83.67 (79.53, 86.29)	to	98.07 (95.01, 101.16)
AUC_0–t_ (µg·h/L)	90.57 (86.91, 93.42)	84.05 (79.96, 86.87)	to	97.61 (94.47, 100.47)
**In silico BE judgment**	7 / 10			

All values are expressed as mean (min, max) in ten trials

## Discussion

Physiologically based absorption modelling performed by GastroPlus has been used to address several aspects of drug development, as reported in previous studies [6–10]. Our study first predicted intestinal absorption of atorvastatin tablets using a compartmental PK model based on Gastroplus software. Moreover, we compared the effect of in vitro dissolution behaviours in different media of atorvastatin tablets on in vivo PK outcomes. We found that in vitro dissolution in three media has similar PK characteristics. Lastly, the predicted results using dissolution profiles in pH 4.5 media showed that the test formulation is bioequivalent to the reference.

The prediction of intestinal absorption of atorvastatin using a compartmental PK model based on Gastroplus has not been found. Our study built an integrated mechanistic compartmental model to predict the PK of atorvastatin based on the data from literature or the ADMET Predictor. Any significant input change to fit the observed data should be adequately considered. Although significant strides have been made in understanding the processes of drug absorption, there are still several unknowns about the intestine. The coefficients of ASF using regional absorption model in GastroPlus can scale the effective permeability of different GI tract sections. It has been frequently observed that for some drugs, the ASFs in the caecum or colon needed to be adjusted to better fit the observed PK [24, 25]. In this study, we found that increasing the ASF in the duodenum and jejunum can obviously increase the C_max_, resulting better fitting of the observed value. This may be due to the significant absorption in the duodenum and ileum for atorvastatin.

In vitro/in vivo correlation (IVIVC) has been developed to reproduce in vivo dissolution behaviour with an in vitro dissolution profile [26, 27]. Compared to pH 1.2 and pH 6.8 media, the tablets showed a faster dissolution rate in pH 4.5 media. However, the in vitro behaviour of the two formulations in different media did not affect in vivo absorption, demonstrating that the absorption rates and degree of the two formulations were independent of the different dissolution rates in 1.2 and pH 6.8 buffer solutions. This may be due to the high solubility of atorvastatin tablets, which results in a complete dissolution in the intestinal condition.

Demonstrating bioequivalence can be challenging, especially for poorly soluble and hypervariable drugs, due to the highly variable conditions within the human gastrointestinal tract [28]. A single simulation might cause misleading information about a BE outcome. Hence, population virtual bioequivalent simulations are needed to predict the BE outcome with the appropriate number of subjects. Virtual BE has gained attraction and evolved from an academic nicety to a regulatory necessity [29]. This study conducted ten crossover virtual trial simulations with 46 randomly selected subjects in each trial. Considering that variability influences the outcome of the bioequivalent study, the %CV for PK parameters was also changed based on previous clinical data. The predicted results indicated that the reference and test formulations were bioequivalent. However, the accuracy of the population simulation results needs to be further verified by clinical studies.

## Conclusion

In summary, we established in silico models to describe the concentration-time profiles of atorvastatin. Through the population PK simulation using a computer model and virtual bioequivalent analysis, the test formulation showed bioequivalence to the reference formulation. Such modelling can be a potentially helpful tool in the drug development for screening formulations that might be bioequivalent, thus reducing time and costs for pharmaceutical companies.

## Data Availability

The datasets generated and/or analysed during the current study are available from the corresponding author on reasonable request.
